# Selective DNA Demethylation Accompanies T Cell Homeostatic Proliferation and Gene Regulation in Lupus-Prone *lpr* Mice

**DOI:** 10.4049/immunohorizons.2000078

**Published:** 2020-10-23

**Authors:** Christopher D. Scharer, Karen A. Fortner, Julie A. Dragon, Scott Tighe, Jeremy M. Boss, Ralph C. Budd

**Affiliations:** * Department of Microbiology and Immunology, School of Medicine, Emory University, Atlanta, GA 30322; † Vermont Center for Immunology and Infectious Diseases, Department of Medicine, University of Vermont Larner College of Medicine, Burlington, VT 05405; ‡ Vermont Integrative Genomics Resource, University of Vermont Larner College of Medicine, Burlington, VT 05405

## Abstract

Systemic lupus erythematosus (SLE) is characterized by increased DNA demethylation in T cells, although it is unclear whether this occurs primarily in a subset of SLE T cells. The process driving the DNA demethylation and the consequences on overall gene expression are also poorly understood and whether this represents a secondary consequence of SLE or a primary contributing factor. Lupus-prone *lpr* mice accumulate large numbers of T cells with age because of a mutation in Fas (CD95). The accumulating T cells include an unusual population of CD4^−^CD8^−^TCR-αβ^+^ (DN) T cells that arise from CD8^+^ precursors and are also found in human SLE. We have previously observed that T cell accumulation in *lpr* mice is due to dysregulation of T cell homeostatic proliferation, which parallels an increased expression of numerous genes in the DN subset, including several proinflammatory molecules and checkpoint blockers. We thus determined the DNA methylome in *lpr* DN T cells compared with their CD8^+^ precursors. Our findings show that DN T cells manifest discrete sites of extensive demethylation throughout the genome, and these sites correspond to the location of a large proportion of the upregulated genes. Thus, dysregulated homeostatic proliferation in *lpr* mice and consequent epigenetic alterations may be a contributing factor to lupus pathogenesis.

## INTRODUCTION

T cells from patients with systemic lupus erythematosus (SLE) are known to manifest evidence of activation and autoreactivity (1, 2). They also contain increased levels of DNA demethylation, one of the main epigenetic regulators of gene expression (3–5). In addition, certain medications that are known to promote DNA demethylation, such as hydralazine and procainamide, can provoke autoreactivity of T cells and drug-induced lupus (6). However, the mechanism driving DNA demethylation in SLE remains obscure.

Lupus-prone *lpr* mice bear a retroposon disruption of the death receptor Fas (CD95) (7). This results in the accumulation of T cells that would ordinarily undergo programmed cell death during homeostatic proliferation (8). Among the accumulating T cells is a subset of polyclonal CD4^−^CD8^−^TCR-αβ^+^ (DN) T cells that derive from CD8^+^ precursors during homeostatic proliferation (9, 10). This subset is also present in human SLE and derives from CD8^+^ T cells (11–13). We have previously observed that compared with their CD8^+^ precursors, DN T cells from *lpr* mice have upregulated gene expression of numerous immune modulating molecules, including the cytolytic machinery of Fas-ligand, Granzyme B, and perforin, as well as inhibitory molecules such as PD-1, Lag3, and IL-10 (14). Initial analysis of one of these genes, *Pdcd1* (PD-1), which is known to be regulated by DNA methylation (15), revealed extensive demethylation of the 5ʹ regulatory region in DN T cells compared with the CD8^+^ precursors subset (14). Based on these observations, we considered that DNA demethylation may occur more extensively in the genome of *lpr* DN T cells as part of the process of homeostatic proliferation. This might serve in part to explain the particular constellation of genes upregulated in these cells.

## MATERIALS AND METHODS

### Mice

Mice were bred and housed in the Association for Assessment and Accreditation of Laboratory Animal Care International–approved animal facilities of The University of Vermont. Original breeding pairs of B6.MRL-*Fas*^*lpr*^/J (*Fas*^*lpr/lpr*^) mice were obtained from The Jackson Laboratory (Bar Harbor, ME). All mice in these studies were on a C57BL/6 background and were used between 10 and 13 wk of age. All animal studies were conducted in accordance with the policies of The University of Vermont’s Animal Care and Use Committee.

### T cell subset purification

Single-cell suspensions of pooled inguinal, brachial, axillary, cervical, and popliteal lymph nodes were prepared in RPMI 1640 containing 25 mM HEPES, 5% v/v bovine calf serum (HyClone, Logan, UT), 5 × 10^−5^ M 2-ME, 100 U/ml penicillin, and 100 U/ml streptomycin. To isolate T cell subsets by negative selection, lymph node cells were incubated with the appropriate Abs (see below), washed, and then incubated by rocking with goat anti-rat and goat anti-mouse IgG-coated beads (QIAGEN). Ab-coated cells were removed by magnetic depletion. Cell suspensions were incubated with anti–MHC class II (3F12), anti-CD11b (M1/70), anti-NK1.1 (PK136), anti-κ (187.1), and anti-CD4 (GK1.5). To obtain CD8^+^ T cells, anti-CD45R (B220 and RA3GB2) was also added. To isolate DN T cells, anti-CD8 (Tib105) was also included. Purity of cell subsets was examined by flow cytometry in each preparation and was consistently >93%.

### Reduced representation bisulfite sequencing

Genomic DNA was prepared using the Quick gDNA Micro Prep Kit (Zymo Research) according to the manufacturer’s instructions. Bisulfite sequencing libraries were generated as described previously using a custom adapter–primer combination (16, 17). Briefly, genomic DNA was digested separately with MspI and TaqI to enrich for CpG containing DNA and combined with methylated PhiX control DNA (New England Biolabs). The resulting DNA was used as input for the KAPA HyperPrep Kit (Roche Diagnostics) with the following modifications. Adapters for ligation contained fully methylated CpGs. Following ligation, adapter-ligated DNA was bisulfite treated using the EpiTect Bisulfite Kit (QIAGEN), and library amplification was performed using the KAPA HiFi HotStart Uracil+ polymerase (Roche Diagnostics) with custom indexing primers. Final libraries were quality checked on a Bioanalyzer 2100 (Agilent Technologies), pooled at equimolar ratio, and sequenced on an Illumina HiSeq1500/2500 RapidRun using a paired-end 2 × 75 Rapid Run flow cell at the Vermont Integrated Genomic Resource Core.

### Reduced representation bisulfite sequencing analysis

The FASTQ files were quality checked using the Fastx Toolkit v0.0.14, adapter content trimmed using Cutadapt v1.12 (18), and data mapped to the mm10 version of the mouse genome using Bismark v0.13 (19). CpG methylation calls were computed using a custom R pipeline that is available upon request. Bisulfite conversion was assessed using the PhiX-methylated spike in DNA, which showed >99% conversion efficiency for all samples. CpG covered with at least 10 reads in each group were used for all downstream analysis. Differentially methylated loci (DML) were determined using the DSS package (20) and CpG with a false discovery rate–corrected *p* value <0.05 and <20% change in methylation between groups were considered significant. CpG were annotated to the nearest gene transcription start site. To identify transcription factor binding motifs at demethylated DML the HOMER findMotifs-Genome.pl script was used with the -size 400 setting. All data visualization and downstream analysis were performed using custom R scripts that are available upon request. DNA methylation data are available from the Gene Expression Omnibus under accession https://www.ncbi.nlm.nih.gov/geo/query/acc.cgi?acc=GSE155293.

### Meta-analysis with microarray data

To determine to what degree the most demethylated genes were part of the set of genes upregulated in DN T cells compared with CD8^+^ T cells, DNA methylation data were integrated with our previous microarray analysis (14). A total of 968 genes were both upregulated and demethylated DN T cells. With this set, Gene Ontology (GO) term enrichment and pathway analysis was conducted using Partek Genomics Suite, version 7.18 (Partek, St. Louis, MO). In addition, functional cluster and pathway analysis was performed using both National Institutes of Health (NIH) Database for Annotation, Visualization, and Integrated Discovery (https://david.ncifcrf.gov) and Ingenuity Pathways Analysis (Ingenuity Systems, www.ingenuity.com). The DML that mapped to the 968 upregulated genes was selected, resulting in 7649 CpG. Among those, 7267 DML were demethylated in the DN subset compared with the CD8^+^ subset.

### Meta-analysis with assay for transposase-accessible chromatin sequencing data

To compute the overlap of DML and accessible chromatin regions, we analyzed previously defined assay for transposase-accessible chromatin sequencing (ATAC-seq) data (accession no. GSE83081) from unstimulated splenic naive CD8^+^ T cells and splenic effector CD8^+^ T cells at day 8 following lymphocytic choriomeningitis virus Armstrong infection (day 8 effector) that were positive for the gp33, gp276, and np396 tetramers (21). A set of accessible regions was assembled by merging the peaks from naive and day 8 effector samples using the HOMER mergePeaks function and converted to a bed file using pos2bed.pl (22). Because the ATAC-seq data were mapped to the mm9 mouse genome, all DML coordinates were converted mm9 using the UCSC Genome Browser liftover tool (23). The overlap of DML and ATAC-seq regions was computed using the bedtools window function with -w as the indicated distance (24). To determine overlap significance, the ATAC-seq regions were randomly shuffled across the genome using the bedtools shuffle command 1000 times and overlap with DML calculated as above for each distance window. The *p* values were calculated as the number of times the permuted overlap was greater than the observed overlap divided by the number of permutations (1000), with *p* values <0.001 resulting from zero permutations being greater than the observed overlap. For plotting ATAC-seq data, the mm9 bigWig tracks were converted to mm10 using the bwtool (25) and the mm9ToMm10.over.chain.gz UCSC Genome Browser chain file.

## RESULTS

### Discrete genome demethylation of lpr DN T cells

**FIGURE 1.**
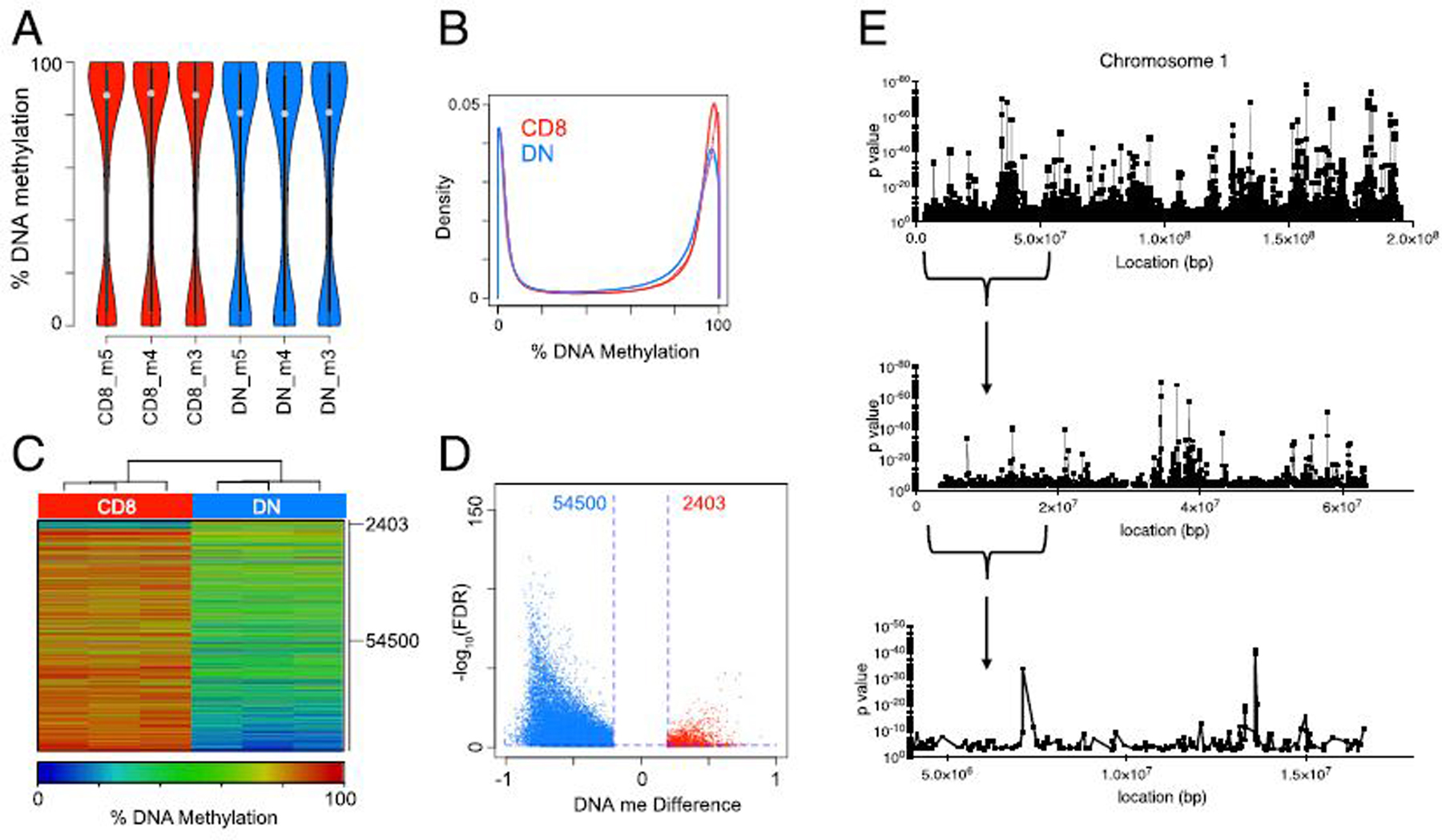
*Lpr* DN T cells have a demethylated genome relative to precursor CD8^+^ T cells. DN and CD8^+^ T cell subsets were purified from B6-*lpr* mice on three separate occasions, using three mice per purification. DNA was extracted and subjected to RRBS and processed as described in the Materials and Methods. **(A)** Violin plots showing the distribution of DNA methylation at all 2.35 × 10^6^ CpG with ×10 coverage. For violin plot gray dots represent the mean and black bars the first and third interquartile ranges. **(B)** Density plots of the distribution of DNA methylation for each sample at the CpG from (A). **(C)** Heatmap of 56,903 DML showing the percentage of DNA methylation for each sample. Ninety-six percentage of methylation differences reflect a loss of methylation in DN. **(D)** Volcano plot showing the change in DNA methylation versus significance for all DML from (B). **(E)** Plot showing the *p* values for all detected CpGs in *lpr* DN T cells compared with CD8^+^ T cells. Three different resolutions are shown for the indicated locations on chromosome 1. The *x*-axis denotes the base pair coordinates of each window on chromosome 1.

DNA from DN T cells and the precursor CD8^+^ T cells of B6-*lpr* mice were subjected to reduced representation bisulfite sequencing (RRBS) (17). B6-*lpr* mice were chosen over MRL-*lpr* mice, as B6-*lpr* mice develop only minimal if any lupus manifestations, allowing better separation of the epigenetic findings from potential confounding processes driven by autoimmune disease. Three separate purifications of both Tcell subsets were made using a pool of three mice in each purification. A total of 2,350,129 CpG with a 10× coverage across both groups of T cells was used for downstream analysis, representing 11% of the roughly 22 million murine CpG. Analysis of the mean DNA methylation levels in each subset revealed that the DN subset had ~2.5% greater demethylation globally compared with the CD8^+^ T cell precursors (Fig. 1A, 1B).

Analysis of significant DML revealed 56,903 CpG (2.4% of total covered) that displayed >20% change in DNA methylation with a false discovery rate <0.05 (Supplemental Table I). Intriguingly, 54,500 (96%) of the DML between these two T cell subsets reflected a loss of DNA methylation in the DN subset, which was highly consistent and significant across the three separate experiments (Fig. 1C, 1D). Consistent with the change in distribution, many of the CpG were close to 100% methylated in all CD8^+^ T cell alleles and shifted to low-intermediate levels of methylation in DN T cells. Of particular interest was that multiple DML clustered at discrete genomic regions, suggesting the changes in DNA methylation were not random. For example, significant changes in DNA methylation along chromosome 1 cluster when viewed at different base pair resolutions (Fig. 1E). Findings for all chromosomes are provided in Gene Expression Omnibus under accession GSE155293. These data demonstrate that DN T cells display wide-ranging DNA demethylation but at discrete loci.

### Overlap of demethylation sites and upregulated genes in DN T cells

**FIGURE 2.**
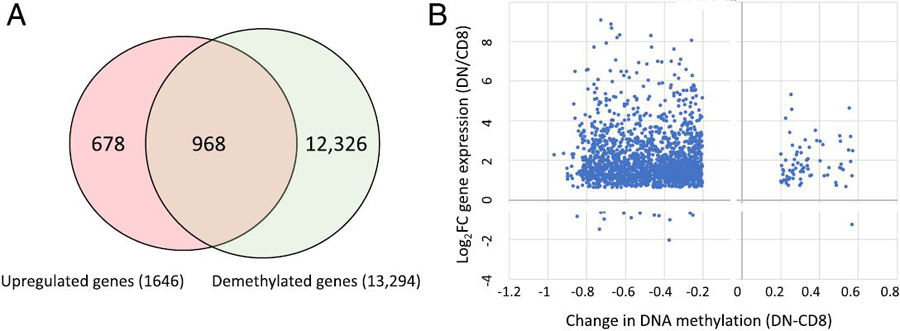
Overlap of upregulated genes in DN T cells with demethylation sites. **(A)** The genome location of 1646 upregulated genes in *lpr* DN T cells overlayed with the sites of DNA demethylation. **(B)** Plot of change in DNA methylation for all DML (*x*-axis) versus the change in gene expression for genes in (A) upregulated in *lpr* DN T cells.

We previously performed gene expression profiling for DN T cells and CD8^+^ T cells from B6-*lpr* mice and noted 1646 genes that were significantly upregulated in DN T cells (14). Among the upregulated genes was *Pdcd1*, and the finding that this locus was also demethylated suggested there might be a wider correlation of DNA methylation changes and gene expression of other upregulated genes in DN T cells. Therefore, DML were assigned to genes by annotating to the nearest transcriptional start site. Strikingly, 968 of the 1646 upregulated genes mapped to DML that lost DNA methylation in the DN subset (Fig. 2A). Given that many of these genes are upregulated in DN T cells, the change in gene expression was correlated with the change in DNA methylation for each DML that mapped to a gene with significant gene expression changes. Consistent with a repressive role for DNA methylation, this analysis revealed an inverse relationship between the two datasets, with the majority of the demethylated loci mapping to genes that gained gene expression (Fig. 2B, top left quadrant).

**FIGURE 3.**
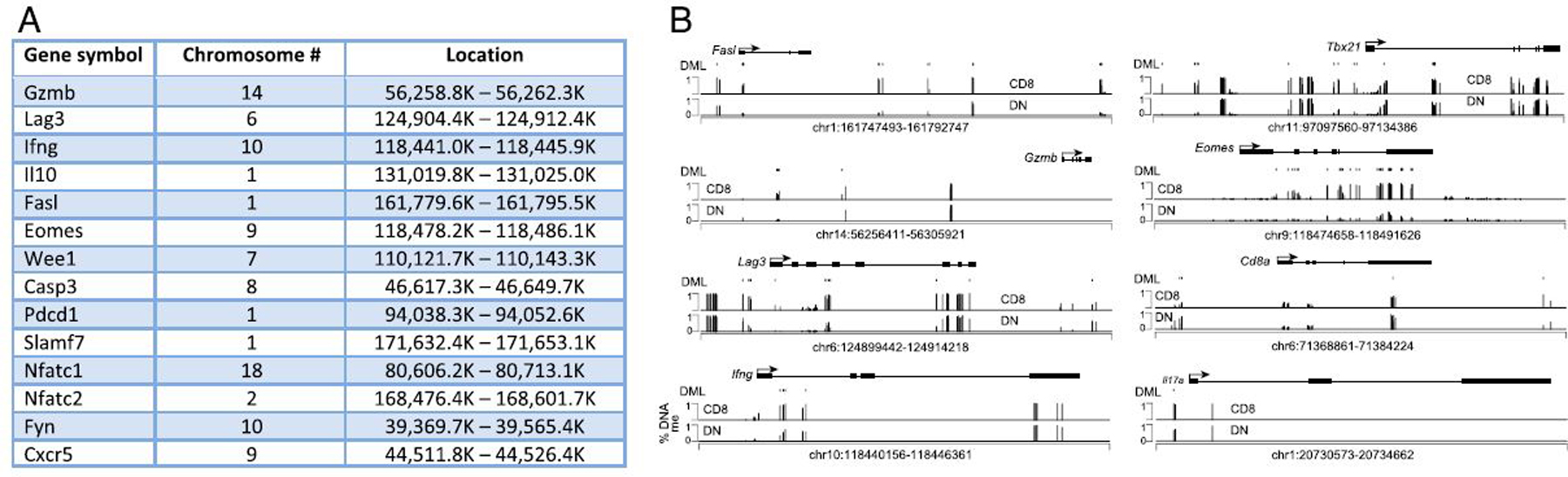
DNA demethylation in DN T cells at sites of immune regulatory genes. **(A)** List of immune regulatory genes that are both upregulated and demethylated in DN T cells. **(B)** Genome plot of the *Fasl*, *Gzmb*, *Lag3*, *Ifng*, and *Eomes* showing the location and percent DNA methylation of DML of CD8^+^ versus DN T cells for the indicated gene and surrounding genomic loci.

Several immune-related genes were both upregulated and located near demethylation sites, including *Nfatc1*, *Nfatc2*, *Fyn*, *Eomes*, *Ifng*, *Il10*, *Fasl*, *Cxcr5*, *Pdcd1*, *Lag3*, *Slamf7*, and *Gzmb*, among others (Fig. 3A). Indeed, multiple demethylated DML can be visualized in the DN subset within the loci for *Fasl*, *Gzmb*, *Lag3*, *Ifng*, Tbx21, *Eomes*, *Cd8a*, and *Il17a* (Fig. 3B).

A GO term enrichment and Kyoto Encyclopedia of Genes and Genomes pathway analysis was performed using this set of 968 upregulated genes to determine if there was an enrichment for specific cellular functions. Among the enriched GO terms were molecular mechanisms of cancer, PI3K signaling, PTEN signaling, and cell death and survival (Table I). The leading Kyoto Encyclopedia of Genes and Genomes pathway was TCR signaling (Supplemental Table I). This was of interest given that the DN T cells arise from CD8^+^ precursors during repeated rounds of homeostatic proliferation, which requires recurrent TCR stimulation by autologous MHC/peptide complexes (26). Additionally, the upregulated genes were ranked according to the number of demethylated DML mapping to each gene (Supplemental Table II). The gene with the most DML and highest ranking was *Foxp1*, which is a known regulator of quiescence in T cells (27, 28). This is consistent with earlier observations that *lpr* DN T cells are small senescent cells that do not proliferate when activated in vitro (29, 30).

### DML occur near regions of accessible chromatin in effector CD8^+^ T cells

**FIGURE 4.**
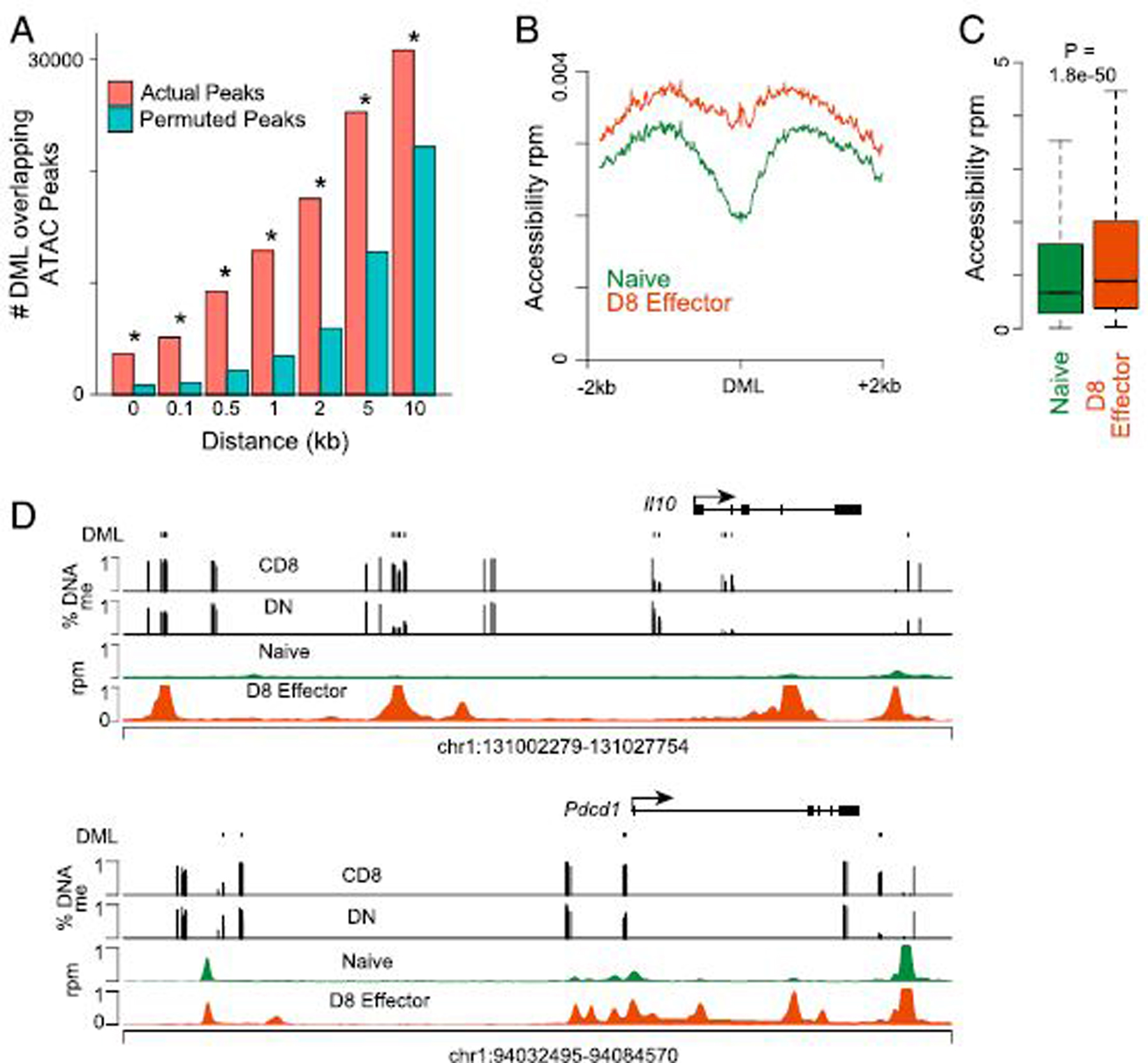
DML in DN T cells are located proximal to accessible *cis*-regulatory elements in CD8^+^ T cells. **(A)** Bar plots showing the number of DML of that map to a peak of accessible chromatin defined in naive or effector CD8^+^ T cells or a set of randomly shuffled regions of equal size at each indicated distance. The percentage of all DML that are matched at each distance is indicated. ATAC-seq data were previously described (21). *p < 0.001, based on 1000 random permutations. **(B)** Histogram showing the accessibility in naive versus day 8 effector CD8^+^ T cells for 2 kb either side of DML. **(C)** Box plot quantitating the accessibility from (B). The *p* value was calculated by two-tailed Student *t* test. **(D)** Genome plot showing the location and percentage DNA methylation for DML of CD8^+^ versus DN T cells and ATAC-seq signal in naive and day 8 effector CD8^+^ T cells for *Il10* and *Pdcd1* loci.

Analysis of the gene expression changes for DNA methyltransferases and demethylases revealed that only *Dnmt3b* was significantly different (Table II), suggesting other mechanisms might explain the observed DNA methylation changes. Analogous to the development of DN T cells from CD8^+^ precursors during homeostatic proliferation, the differentiation of CD8^+^ T cells from naive to effector cells involves extensive remodeling of accessible chromatin and DNA methylation at *cis*-regulatory regions (21, 31). The observed clustering of DML and their proximity to genes that were remodeled during effector CD8^+^ T cell differentiation suggested that the DNA methylation changes may be associated with similar *cis*-regulatory elements. Therefore, using a range of distance windows, the overlap of each DML to a region of accessible chromatin in naive or day 8 effector CD8^+^ T cells responding to lymphocytic choriomeningitis virus from a previous study (21) was calculated. An increasing overlap of DML and accessible regions was observed, which was greater than a set of randomly shuffled sequences, with 16% within 500 bp and 54% within 10 kb (Fig. 4A). Analysis of the accessibility in the 4 kb surrounding DML demonstrated a significant gain in accessibility in day 8 effector CD8^+^ T cells over naive CD8^+^ T cells (Fig. 4B, 4C). For example, the *Il10* and *Pdcd1* (PD-1) loci contained DML that both overlapped and occurred in proximity to regions that gain accessibility in day 8 effector CD8^+^ T cells (Fig. 4D). Thus, DNA methylation occurs in proximity to *cis*-regulatory elements that gain accessibility in effector CD8^+^ T cells.

### DML are enriched for AP-1, T-BET, and EGR transcription factor binding motifs

**FIGURE 5.**
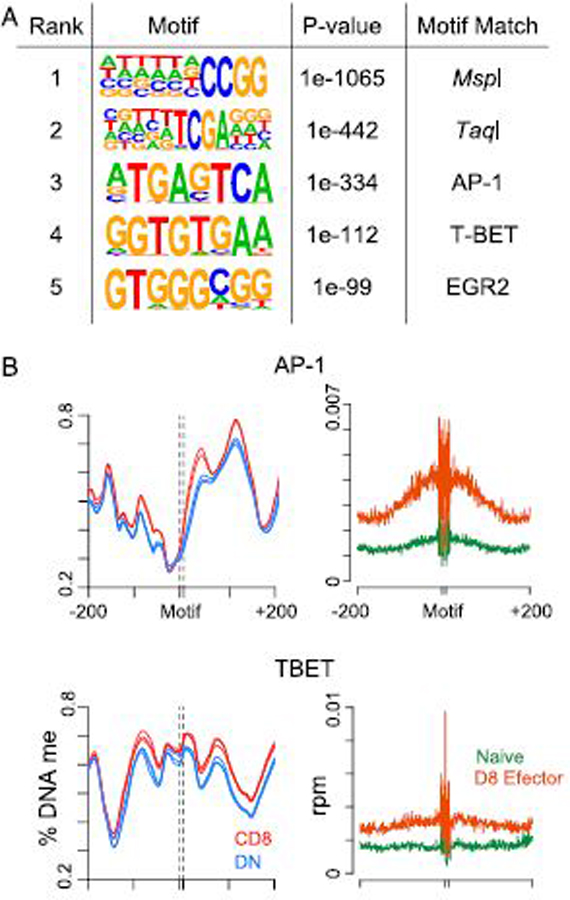
Enrichment of effector transcription factors at demethylated DML of DN T cells. **(A)** Table showing the enriched motif, *p* value, and matched transcription factor in the 200 bp surrounding demethylated DML. **(B)** Histogram showing the percentage DNA methylation (left) in DN and CD8^+^ T cell subsets and chromatin accessibility (right) in naive and day 8 effector CD8^+^ T cells at the 200 bp either side of AP-1 and T-BET motifs.

Regions that demonstrate dynamic DNA methylation during CD8^+^ T cell differentiation are enriched for transcription factor binding motifs that play important roles in CD8^+^ T cell fate and function (31). To determine if similar mechanisms occurred at the demethylated CpG in DN T cells, the surrounding 200 bp of each DML was searched for enriched transcription factor binding motifs using HOMER (22). As expected, the top two scoring enriched motifs contained sites for the *MspI* (CCGG) and *TaqI* (TCGA) restriction enzymes used in the RRBS assay (Fig. 5A). The next top scoring motifs were for the transcription factors AP-1, T-BET, and EGR2, all of which are involved in CD8^+^ T cell effector function (32–34). To further examine the epigenetic changes, the levels of DNA methylation and accessibility were computed for the 400 bp surrounding both AP-1 and T-BET motifs. Both motifs demonstrated a loss in DNA methylation in DN T cells versus CD8^+^ T cells (Fig. 5B). Consistent with this finding, each motif showed a higher level of accessibility in day 8 effector compared with naive CD8^+^ T cells. These data suggest that DML in proximity to transcription factor binding sites drive an effector program as CD8^+^ T cells transition to DN T cells during homeostatic proliferation.

## DISCUSSION

The current findings reveal an epigenetic program of global, yet highly selective, DNA demethylation accompanied by upregulation of numerous genes, both of which accompany T cell homeostatic proliferation in *lpr* mice. Unlike MRL-*lpr* mice, B6-*lpr* mice develop very little autoimmune disease with age. We thus intentionally performed these studies using B6-*lpr* mice to separate the epigenetic changes that accompany T cell homeostatic proliferation from confounding factors that might be secondary to autoimmune disease. The findings therefore may be more broadly applicable to T cell homeostatic proliferation also in wild-type mice. In fact, we have previously observed that successive rounds of T cell homeostatic proliferation in B6 wild-type mice parallel gene expression changes nearly identical to B6-*lpr* T cells, and a DN subset is also observed in B6 wild-type mice (14). Conceivably, epigenetic regulation of a similar constellation of genes may occur in wild-type mice during T cell homeostatic proliferation but are enhanced in lupus-prone mice.

T cells from SLE patients bear demethylated DNA (3–5), but the underlying cellular process regulating this was not fully defined. It was also not clear whether the demethylation occurred primarily in a subset of SLE T cells or globally in all T cells. Most of the studies on DNA methylation in lupus T cells have been conducted on CD4^+^ T cells, whereas the current study examined DNT cells and their precursor CD8^+^ Tcells. Nonetheless, there are remarkable similarities in upregulated gene expression between SLE CD4^+^ T cells and *lpr* DN T cells, including *ITGAL* (CD11a), *CD40LG* (CD40-ligand), *IFNG* (IFN-γ), *RFR1* (perforin), *GADD45A*, and *CXCR3* (5), suggesting a possible common mechanism. Because DN T cells also occur in human SLE (2, 11), conceivably this subset in human SLE may also bear a substantial portion of the DNA demethylation in SLE T cells. Although most humans with SLE do not bear *Fas* gene mutations, it is possible that mechanisms operative in SLE might accelerate T cell homeostatic proliferation, resulting in a similar epigenetic program.

Recently, a study of PBMC from 17 monozygotic and dizygotic twin pairs discordant for SLE revealed extensive demethylation at 807 CpG sites corresponding to 49 genes in the affected twin compared with their healthy twin (35). This was not observed in twinsdiscordant forrheumatoid arthritis or diabetes mellitus. This study also found that the SLE patients had reduced mRNAlevels of the DNA methyltransferase, *Dnmt3b*, which we also observed to be decreased in *lpr* DN T cells (Table II). A further similarity is that several of the immune genes that were both demethylated and upregulated in the SLE twin study were also demethylated and upregulated in *lpr* DN T cells, including *Il10*, *Grb10*, *Gfi1*, *Padi4*, *Cd9*, and *Aim2* (14, 35). In addition, DNA demethylation of a particular gene, *Tnfsf7* (CD70), has been observed in 16-wk autoimmune MRL-*lpr* mice compared with 5-wk mice prior to onset of autoimmunity (36). This was associated with a reduction in levels of DNMT1 (36).

The current findings also have striking similarities to our recent epigenetic and transcriptional analyses of B cells in human SLE (16). A subpopulation of IgD^−^CD27^−^CXCR7^−^CD11c^+^ (DN2) B cells is expanded in SLE and has been linked to disease (37). DN2 B cells also share some similarity with autoantibody-associated B cells described in mice (18, 38). Interestingly, the AP-1, T-BET, and EGR transcription factor binding motifs that were observed in this study are also enriched in the SLE-specific accessible chromatin (16). *Lpr* DN T cells express high levels of the AP-1 complex (39) and manifest high levels of *Ifng* (14). Additionally, the gene expression profiling showed a parallel dysregulation of similar gene networks, among them regulation of cell cycling, glycolysis, oxidative phosphorylation, and apoptosis. This parallels the known high levels of both glycolysis and oxygen consumption by *lpr* DN T cells (40, 41). Additional common groups of upregulated genes included TCR signaling and protein phosphorylation. This is also consistent with the known constitutive phosphorylation of many signaling proteins in *lpr* DN Tcells, including several TCR signaling components, such as CD3ζ and Fyn (42, 43).

In addition to the numerous upregulated genes related to cell cycling and cytokine production, both *lpr* DN T cells and SLE DN2 B cells also manifest upregulation of the cell cycle checkpoint blocker PD-1 (*Pdcd1*) and IL-10, and the sites of both genes correspond to sites of demethylation (16). It is of interest that homeostatically repopulating B cells following depletion with rituximab also express high levels of IL-10 (44). Collectively, these findings may suggest that *lpr* DN T cells, SLE DN2 B cells, and B cells following rituximab share a common epigenetic and transcriptional program linked to increased homeostatic proliferation.

A recent study showed that treatment of MRL-*lpr* mice with 5-azacytidine (5-Aza), a chemical analogue of cytidine that inhibits DNA methylation, enhanced autoimmunity, whereas targeting 5-Aza to only CD4+ or CD8 T cells alleviated disease (45). The reason for this seeming paradox was not fully explored, but 5-Aza also inhibits DNA replication, which could reduce the adenopathy of these mice when targeted to T cells, a feature not described in the study.

There may be several reasons for the progressive demethylation in *lpr* DN T cells. Expression of the DNA methyltransferase, *Dnmt3b*, is reduced in this subset compared with the CD8^+^ precursors, which could contribute to this genotype. An alternative is that rapidly proliferating cells can exceed the ability of DNA methyltransferases to keep pace with the rapid DNA replication rate (46). Consistent with this, we have previously observed that the *lpr* DN T cells have undergone very rapid proliferation in vivo, with up to 18% replicating during a single 24-h period, as defined by BrdU uptake (14, 26). An additional possibility is oxidative stress–induced demethylation. T cells from patients with active lupus manifest increased mitochondrial oxidative phosphorylation and reactive oxygen species (ROS) (47, 48). Oxidative stress of CD4^+^ T cells results in decreased levels of DNMT1, DNA demethylation, and upregulation in expression of several genes (4). Moreover, adoptive transfer of oxidant-treated CD4^+^ T cells into syngeneic mice caused anti-dsDNA Ab and glomerulonephritis (49). Consistent with this, as noted earlier, *lpr* DN T cells manifest increased oxygen consumption compared with CD8^+^ precursor cells in part because of increased mitochondrial mass that parallels homeostatic proliferation (41).

Several of the demethylated and upregulated genes in *lpr* DN T cells are associated with inflammation (*FasL*, *GzmB*, *Prf1*, and *Ifng*) and immune exhaustion (*Pdcd1* and *Lag3*). This might help explain the clinical immunology paradox of individuals with immunodeficiency syndromes either genetically, from chemotherapy, or because of HIV, which suddenly develop autoimmune syndromes. A dramatic example is the sudden onset of psoriasis and psoriatic arthritis in HIV^+^ individuals (50). Conceivably, the lymphopenia in these conditions could lead to accelerated T cell homeostatic proliferation with resulting upregulation of inflammatory molecules. Conversely, SLE patients, bearing a seemingly overactive immune system, are nonetheless prone to infections and often respond poorly to vaccinations (51). It is possible that upregulation of PD-1 and Lag3 in SLE T cells renders them less responsive to new activation.

In summary, homeostatic proliferation of T cells manifests a broad program of both genetic and metabolic changes that could influence immune function and inflammatory autoimmune conditions. This includes the increased mitochondrial mass and size in T cells undergoing homeostatic proliferation (41). This contributes to high oxygen consumption rates, ROS production that can induce oligomerization of MAVS, and increased type I IFN (41, 52). ROS and cell proliferation may also contribute to DNA demethylation (4). The current studies, thus, expand our knowledge of functional modifications during T cell homeostatic proliferation to reveal an epigenetic program of DNA demethylation at selective sites throughout the genome, contributing to upregulation of several immune response genes.

## Supplementary Material

Supplemental Table II

Supplemental Table I

## Figures and Tables

**TABLE I.** GO pathway analysis of genes upregulated in DN T cells that also localize to demethylation sites

Name	*p* Value	Overlap
Top canonical pathways		
Molecular mechanisms of cancer	2.33 × 10^−8^	10.9% (43/394)
Axonal guidance signaling	1.85 × 10^−5^	8.8% (40/457)
PI3K signaling	2.22 × 10^−5^	13.2% (18/136)
Epithelial adherens junction signaling	2.49 × 10^−5^	12.7% (19/150)
PTEN signaling	2.61 × 10^−5^	13.6% (17/125)
Name	*p* Value	No. of Molecules

Top molecular and cellular functions		
Cell death and survival	6.07 × 10^−7^ to 1.86 × 10^−34^	415
Cellular assembly and organization	3.22 × 10^−7^ to 1.25 × 10^−30^	263
Cellular function and maintenance	4.37 × 10^−5^ to 1.25 × 10^−30^	382
Cellular movement	7.66 × 10^−7^ to 9.47 × 10^−29^	292
Cellular development	6.81 × 10^−7^ to 1.64 × 10^−23^	416

**TABLE II.** Expression of DNA methyltransferases and demethylases in *lpr* DN compared with CD8^+^ T cells

Gene	Log Fold Change (DN − CD8^+^)	*p* Value
*Dnmt1*	−0.012	0.655
*Dnmt3a*	0.318	0.131
*Dnmt3b*	−1.322	2.17 × 10^−6^
*Tet1*	0.261	0.257
*Tet2*	0.084	0.392

## Abbreviations used in this article:

ATAC-seq: assay for transposase-accessible chromatin sequencing
5-Aza: 5-azacytidine
DML: differentially methylated locus
DN: CD4^−^CD8^−^TCR-αβ^+^
GO: Gene Ontology
NIH: National Institutes of Health
ROS: reactive oxygen species
RRBS: reduced representation bisulfite sequencing
SLE: systemic lupus erythematosus

## References

[1] Shlomchik MJ, Craft JE, and Mamula MJ 2001. From T to B and back again: positive feedback in systemic autoimmune disease. Nat. Rev. Immunol 1: 147–153. 11905822 10.1038/35100573

[2] Tsokos GC, Mitchell JP, and Juang YT 2003. T cell abnormalities in human and mouse lupus: intrinsic and extrinsic. Curr. Opin. Rheumatol 15: 542–547. 12960478 10.1097/00002281-200309000-00004

[3] Richardson B, Scheinbart L, Strahler J, Gross L, Hanash S, and Johnson M 1990. Evidence for impaired T cell DNA methylation in systemic lupus erythematosus and rheumatoid arthritis. Arthritis Rheum 33: 1665–1673. 2242063 10.1002/art.1780331109

[4] Li Y, Gorelik G, Strickland FM, and Richardson BC 2014. Oxidative stress, T cell DNA methylation, and lupus. Arthritis Rheumatol 66: 1574–1582. 24577881 10.1002/art.38427 PMC4141415

[5] Zhang Y, Zhao M, Sawalha AH, Richardson B, and Lu Q 2013. Impaired DNA methylation and its mechanisms in CD4(+)T cells of systemic lupus erythematosus. J. Autoimmun 41: 92–99. 23340289 10.1016/j.jaut.2013.01.005

[6] Cornacchia E, Golbus J, Maybaum J, Strahler J, Hanash S, and Richardson B 1988. Hydralazine and procainamide inhibit T cell DNA methylation and induce autoreactivity. J. Immunol 140: 2197–2200. 3258330

[7] Adachi M, Watanabe-Fukunaga R, and Nagata S 1993. Aberrant transcription caused by the insertion of an early transposable element in an intron of the Fas antigen gene of lpr mice. Proc. Natl. Acad. Sci. USA 90: 1756–1760. 7680478 10.1073/pnas.90.5.1756 PMC45958

[8] Fortner KA, and Budd RC 2005. The death receptor Fas (CD95/APO-1) mediates the deletion of T lymphocytes undergoing homeostatic proliferation. J. Immunol 175: 4374–4382. 16177078 10.4049/jimmunol.175.7.4374

[9] Landolfi MM, Van Houten N, Russell JQ, Scollay R, Parnes JR, and Budd RC 1993. CD2-CD4-CD8-lymph node T lymphocytes in MRL lpr/lpr mice are derived from a CD2+CD4+CD8+ thymic precursor. J. Immunol 151: 1086–1096. 7687614

[10] Mixter PF, Russell JQ, Durie FH, and Budd RC 1995. Decreased CD4-CD8-TCR-alpha beta + cells in lpr/lpr mice lacking beta 2-microglobulin. J. Immunol 154: 2063–2074. 7868883

[11] Crispín JC, Oukka M, Bayliss G, Cohen RA, Van Beek CA, Stillman IE, Kyttaris VC, Juang YT, and Tsokos GC 2008. Expanded double negative T cells in patients with systemic lupus erythematosus produce IL-17 and infiltrate the kidneys. J. Immunol 181: 8761–8766. 19050297 10.4049/jimmunol.181.12.8761 PMC2596652

[12] Shivakumar S, Tsokos GC, and Datta SK 1989. T cell receptor alpha/beta expressing double-negative (CD4-/CD8-) and CD4+ T helper cells in humans augment the production of pathogenic anti-DNA auto-antibodies associated with lupus nephritis. J. Immunol 143: 103–112. 2525144

[13] Crispín JC, and Tsokos GC 2009. Human TCR-alpha beta+ CD4-CD8- T cells can derive from CD8+ T cells and display an inflammatory effector phenotype. J. Immunol 183: 4675–4681. 19734235 10.4049/jimmunol.0901533 PMC2878279

[14] Fortner KA, Bond JP, Austin JW, Boss JM, and Budd RC 2017. The molecular signature of murine T cell homeostatic proliferation reveals both inflammatory and immune inhibition patterns. J. Autoimmun 82: 47–61. 28551033 10.1016/j.jaut.2017.05.003 PMC5902411

[15] Youngblood B, Oestreich KJ, Ha SJ, Duraiswamy J, Akondy RS, West EE, Wei Z, Lu P, Austin JW, Riley JL, 2011. Chronic virus infection enforces demethylation of the locus that encodes PD-1 in antigen-specific CD8(+) T cells. Immunity 35: 400–412. 21943489 10.1016/j.immuni.2011.06.015 PMC3183460

[16] Scharer CD, Blalock EL, Mi T, Barwick BG, Jenks SA, Deguchi T, Cashman KS, Neary BE, Patterson DG, Hicks SL, 2019. Epigenetic programming underpins B cell dysfunction in human SLE. Nat. Immunol 20: 1071–1082. 31263277 10.1038/s41590-019-0419-9 PMC6642679

[17] Barwick BG, Scharer CD, Bally APR, and Boss JM 2016. Plasma cell differentiation is coupled to division-dependent DNA hypomethylation and gene regulation. Nat. Immunol 17: 1216–1225. 27500631 10.1038/ni.3519 PMC5157049

[18] Hao Y, O’Neill P, Naradikian MS, Scholz JL, and Cancro MP 2011. A B-cell subset uniquely responsive to innate stimuli accumulates in aged mice. Blood 118: 1294–1304. 21562046 10.1182/blood-2011-01-330530 PMC3152496

[19] Krueger F, and Andrews SR 2011. Bismark: a flexible aligner and methylation caller for bisulfite-seq applications. Bioinformatics 27: 1571–1572. 21493656 10.1093/bioinformatics/btr167 PMC3102221

[20] Park Y, and Wu H 2016. Differential methylation analysis for BS-seq data under general experimental design. Bioinformatics 32: 1446–1453. 26819470 10.1093/bioinformatics/btw026 PMC12157722

[21] Scharer CD, Bally APR, Gandham B, and Boss JM 2017. Cutting edge: chromatin accessibility programs CD8 T cell memory. J. Immunol 198: 2238–2243. 28179496 10.4049/jimmunol.1602086 PMC5341694

[22] Heinz S, Benner C, Spann N, Bertolino E, Lin YC, Laslo P, Cheng JX, Murre C, Singh H, and Glass CK 2010. Simple combinations of lineage-determining transcription factors prime cisregulatory elements required for macrophage and B cell identities. Mol. Cell 38: 576–589. 20513432 10.1016/j.molcel.2010.05.004 PMC2898526

[23] Kent WJ, Sugnet CW, Furey TS, Roskin KM, Pringle TH, Zahler AM, and Haussler D 2002. The human genome browser at UCSC. Genome Res 12: 996–1006. 12045153 10.1101/gr.229102 PMC186604

[24] Quinlan AR, and Hall IM 2010. BEDTools: a flexible suite of utilities for comparing genomic features. Bioinformatics 26: 841–842. 20110278 10.1093/bioinformatics/btq033 PMC2832824

[25] Pohl A, and Beato M 2014. bwtool: a tool for bigWig files. Bioinformatics 30: 1618–1619. 24489365 10.1093/bioinformatics/btu056 PMC4029031

[26] Fortner KA, Lees RK, MacDonald HR, and Budd RC 2011. Fas (CD95/APO-1) limits the expansion of T lymphocytes in an environment of limited T-cell antigen receptor/MHC contacts. Int. Immunol 23: 75–88. 21266499 10.1093/intimm/dxq466 PMC3030730

[27] Feng X, Wang H, Takata H, Day TJ, Willen J, and Hu H 2011. Transcription factor Foxp1 exerts essential cell-intrinsic regulation of the quiescence of naive T cells. Nat. Immunol 12: 544–550. 21532575 10.1038/ni.2034 PMC3631322

[28] Ren J, Han L, Tang J, Liu Y, Deng X, Liu Q, Hao P, Feng X, Li B, Hu H, and Wang H 2019. Foxp1 is critical for the maintenance of regulatory T-cell homeostasis and suppressive function. PLoS Biol 17: e3000270. 31125332 10.1371/journal.pbio.3000270 PMC6534289

[29] Clements JL, Winslow G, Donahue C, Cooper SM, Allison JP, and Budd RC 1993. Co-stimulation via CD28 induces activation of a refractory subset of MRL-lpr/lpr T lymphocytes. Int. Immunol 5: 1451–1460. 7903158 10.1093/intimm/5.11.1451

[30] Clements JL, Wolfe J, Cooper SM, and Budd RC 1994. Reversal of hyporesponsiveness in lpr CD4-CD8- T cells is achieved by induction of cell cycling and normalization of CD2 and p59fyn expression. Eur. J. Immunol 24: 558–565. 7510235 10.1002/eji.1830240310

[31] Scharer CD, Barwick BG, Youngblood BA, Ahmed R, and Boss JM 2013. Global DNA methylation remodeling accompanies CD8 T cell effector function. J. Immunol 191: 3419–3429. 23956425 10.4049/jimmunol.1301395 PMC3800465

[32] Chen Y, Zander R, Khatun A, Schauder DM, and Cui W 2018. Transcriptional and epigenetic regulation of effector and memory CD8 T cell differentiation. Front. Immunol 9: 2826. 30581433 10.3389/fimmu.2018.02826 PMC6292868

[33] Sullivan BM, Juedes A, Szabo SJ, von Herrath M, and Glimcher LH 2003. Antigen-driven effector CD8 T cell function regulated by T-bet. Proc. Natl. Acad. Sci. USA 100: 15818–15823. 14673093 10.1073/pnas.2636938100 PMC307651

[34] Du N, Kwon H, Li P, West EE, Oh J, Liao W, Yu Z, Ren M, and Leonard WJ 2014. EGR2 is critical for peripheral näıve T-cell differentiation and the T-cell response to influenza. Proc. Natl. Acad. Sci. USA 111: 16484–16489. 25368162 10.1073/pnas.1417215111 PMC4246296

[35] Javierre BM, Fernandez AF, Richter J, Al-Shahrour F, Martin-Subero JI, Rodriguez-Ubreva J, Berdasco M, Fraga MF, O’Hanlon TP, Rider LG, 2010. Changes in the pattern of DNA methylation associate with twin discordance in systemic lupus erythematosus. Genome Res 20: 170–179. 20028698 10.1101/gr.100289.109 PMC2813473

[36] Sawalha AH, and Jeffries M 2007. Defective DNA methylation and CD70 overexpression in CD4+ T cells in MRL/lpr lupus-prone mice. Eur. J. Immunol 37: 1407–1413. 17429846 10.1002/eji.200636872

[37] Jenks SA, Cashman KS, Zumaquero E, Marigorta UM, Patel AV, Wang X, Tomar D, Woodruff MC, Simon Z, Bugrovsky R, 2018. Distinct effector B cells induced by unregulated toll-like receptor 7 contribute to pathogenic responses in systemic lupus erythematosus. [Published erratum appears in 2020 Immunity 52: 203.] Immunity 49: 725–739.e6. 30314758 10.1016/j.immuni.2018.08.015 PMC6217820

[38] Rubtsov AV, Rubtsova K, Fischer A, Meehan RT, Gillis JZ, Kappler JW, and Marrack P 2011. Toll-like receptor 7 (TLR7)-driven accumulation of a novel CD11c+ B-cell population is important for the development of autoimmunity. Blood 118: 1305–1315. 21543762 10.1182/blood-2011-01-331462 PMC3152497

[39] Clements JL, Cooper SM, and Budd RC 1995. Abnormal regulation of the IL-2 promoter in lpr CD4-CD8- T lymphocytes results in constitutive expression of a novel nuclear factor of activated T cells-binding factor. J. Immunol 154: 6372–6381. 7759875

[40] Secinaro MA, Fortner KA, Collins C, Rincón M, and Budd RC 2019. Glycolysis induces MCJ expression that links T cell proliferation with caspase-3 activity and death. Front. Cell Dev. Biol 7: 28. 30915331 10.3389/fcell.2019.00028 PMC6421275

[41] Fortner KA, Blanco LP, Buskiewicz I, Huang N, Gibson PC, Cook DL, Pedersen HL, Yuen PST, Murphy MP, Perl A, 2020. Targeting mitochondrial oxidative stress with MitoQ reduces NET formation and kidney disease in lupus-prone MRL-*lpr* mice. [Published erratum appears in 2020 Lupus Sci. Med. 7: e000387corr1.] Lupus Sci. Med 7: e000387. 32343673 10.1136/lupus-2020-000387 PMC7199895

[42] Samelson LE, Davidson WF, Morse HC III, and Klausner RD 1986. Abnormal tyrosine phosphorylation on T-cell receptor in lymphoproliferative disorders. Nature 324: 674–676. 2432431 10.1038/324674a0

[43] Katagiri T, Urakawa K, Yamanashi Y, Semba K, Takahashi T, Toyoshima K, Yamamoto T, and Kano K 1989. Overexpression of src family gene for tyrosine-kinase p59fyn in CD4-CD8- T cells of mice with a lymphoproliferative disorder. [Published erratum appears in 1990 Proc. Natl. Acad. Sci. USA 87: 2865.] Proc. Natl. Acad. Sci. USA 86: 10064–10068. 2513573 10.1073/pnas.86.24.10064 PMC298644

[44] Quan C, ZhangBao J, Lu J, Zhao C, Cai T, Wang B, Yu H, Qiao J, and Lu C 2015. The immune balance between memory and regulatory B cells in NMO and the changes of the balance after methylprednisolone or rituximab therapy. J. Neuroimmunol 282: 45–53. 25903728 10.1016/j.jneuroim.2015.03.016

[45] Li H, Tsokos MG, Bickerton S, Sharabi A, Li Y, Moulton VR, Kong P, Fahmy TM, and Tsokos GC 2018. Precision DNA demethylation ameliorates disease in lupus-prone mice. JCI Insight 3: e120880. 10.1172/jci.insight.120880 PMC6141184 30135300

[46] Kagiwada S, Kurimoto K, Hirota T, Yamaji M, and Saitou M 2013. Replication-coupled passive DNA demethylation for the erasure of genome imprints in mice. EMBO J 32: 340–353. 23241950 10.1038/emboj.2012.331 PMC3567490

[47] Gergely P Jr., Grossman C, Niland B, Puskas F, Neupane H, Allam F, Banki K, Phillips PE, and Perl A 2002. Mitochondrial hyperpolarization and ATP depletion in patients with systemic lupus erythematosus. Arthritis Rheum 46: 175–190. 11817589 10.1002/1529-0131(200201)46:1<175::AID-ART10015>3.0.CO;2-H PMC4020417

[48] Gergely P Jr., Niland B, Gonchoroff N, Pullmann R Jr., Phillips PE, and Perl A 2002. Persistent mitochondrial hyperpolarization, increased reactive oxygen intermediate production, and cytoplasmic alkalinization characterize altered IL-10 signaling in patients with systemic lupus erythematosus. J. Immunol 169: 1092–1101. 12097418 10.4049/jimmunol.169.2.1092 PMC4020441

[49] Strickland FM, Li Y, Johnson K, Sun Z, and Richardson BC 2015. CD4(+) T cells epigenetically modified by oxidative stress cause lupus-like autoimmunity in mice. J. Autoimmun 62: 75–80. 26165613 10.1016/j.jaut.2015.06.004 PMC4529773

[50] Arnett FC, Reveille JD, and Duvic M 1991. Psoriasis and psoriatic arthritis associated with human immunodeficiency virus infection. Rheum. Dis. Clin. North Am 17: 59–78. 2041889

[51] Torres-Ruiz J, Mejía-Domínguez NR, Zentella-Dehesa A, Ponce-de-León A, Morales-Padilla SR, Vázquez-Rodríguez R, Alvarado-Lara MR, Reyna-de-la-Garza RA, Tapia-Rodríguez M, Juárez-Vega G, 2019. The systemic lupus erythematosus infection predictive index (LIPI): a clinical-immunological tool to predict infections in lupus patients. Front. Immunol 9: 3144. 30692998 10.3389/fimmu.2018.03144 PMC6340073

[52] Buskiewicz IA, Montgomery T, Yasewicz EC, Huber SA, Murphy MP, Hartley RC, Kelly R, Crow MK, Perl A, Budd RC, and Koenig A 2016. Reactive oxygen species induce virus-independent MAVS oligomerization in systemic lupus erythematosus. [Published erratum appears in 2017 Sci. Signal. 10: eaan5765.] Sci. Signal 9: ra115. 27899525 10.1126/scisignal.aaf1933 PMC5321043

